# On the evaluation of the carbon dioxide solubility in polymers using gene expression programming

**DOI:** 10.1038/s41598-023-39343-8

**Published:** 2023-08-02

**Authors:** Behnam Amiri-Ramsheh, Menad Nait Amar, Mohammadhadi Shateri, Abdolhossein Hemmati-Sarapardeh

**Affiliations:** 1 Department of Petroleum Engineering, Shahid Bahonar University of Kerman, Kerman, Iran; 2 Département Etudes Thermodynamiques, Division Laboratoires, Sonatrach, Boumerdes, Algeria; 3 Department of System Engineering, École de Technologie Supérieur, Montreal, QC Canada; 4 State Key Laboratory of Petroleum Resources and Prospecting, China University of Petroleum (Beijing), Beijing, China

**Keywords:** Energy science and technology, Engineering

## Abstract

Evaluation, prediction, and measurement of carbon dioxide (CO_2_) solubility in different polymers are crucial for engineers in various chemical applications, such as extraction and generation of novel materials. In this paper, correlations based on gene expression programming (GEP) were generated to predict the value of carbon dioxide solubility in three polymers. Results showed that the generated correlations could represent an outstanding efficiency and provide predictions for carbon dioxide solubility with satisfactory average absolute relative errors of 9.71%, 5.87%, and 1.63% for polystyrene (PS), polybutylene succinate-co-adipate (PBSA), and polybutylene succinate (PBS), respectively. Trend analysis based on Henry’s law illustrated that increasing pressure and decreasing temperature lead to an increase in carbon dioxide solubility. Finally, outlier discovery was applied using the leverage approach to detect the suspected data points. The outlier detection demonstrated the statistical validity of the developed correlations. William’s plot of three generated correlations showed that all of the data points are located in the valid zone except one point for PBS polymer and three points for PS polymer.

## Introduction

In the recent years, application of different polymers has become an attractive issue in various industries including the petroleum industry. The fluid adsorption process in different polymers is a vital circumstance in the oil industry concepts such as enhanced oil recovery (EOR)^1–3^, gas separation, imbibition of additives, and foaming processes^4,5^. Carbon dioxide (CO_2_) is one of the most significant gases, which plays a noteworthy role in polymers’ structure, polymer foams, and production properties^4,6^. Also, CO_2_ and supercritical carbon dioxide (SCCO_2_), (a supercritical carbon dioxide is described as a fluid for which both temperature and pressure are higher than critical values) have become one of the most conventional green materials, that have been extensively used in solvent, anti-solvent or a solute in numerous field processing including material synthesis, material modification, foaming processes, polymerization and particle production^7–9^. SCCO_2_ is potentially appealing as a solvent that shows properties that are a mixture of those commonly combined with liquids or gases. CO_2_ solubility is the maximum CO_2_ quantity that can solute in different solutions. Evaluation, prediction, and measurement of CO_2_ solubility in different biodegradable polymers has become notable technology for engineers in various chemical applications such as extraction and generation of novel materials^10–14^. Biodegradable polymers are a particular type of polymers that collapse by bacterial dissolution process to eventuate in natural fluids such as CO_2_ and N_2_. Poly butylene succinate (PBS) and polybutylene succinate-co-adipate (PBSA) are two applicable biodegradable polymers that have been generated by Showa Highpolymer Co. Ltd. and Showa Denko K.K^15,16^.

In order to predict gas solubilities in polymers, especially CO_2_, various experimental, empirical, and theoretical approaches were investigated since 1986. In 1986 and 1993, Shah et al.^17,18^ measured solubility of different gases including CO_2_ in silicone polymers at pressures up to 26 atmosphere and temperature values of 10, 35, and 55 °C. In 1994, Li et al.^19^ predicted the solubility of CO_2_ in amine systems. They considered binary and ternary mixtures containing three solvents, namely mono-ethanolamine (MEA), methyl-diethanolamine (MDEA), and water (H_2_O). They used temperature in a range of 0–225 °C. They modeled CO_2_ solubility in amine mixtures as a function of temperature. Two years later, Sato et al.^20^ investigated solubility of CO_2_ and N_2_ in polystyrene under high pressure and temperature conditions. They measured gas solubility at pressures up to 20 MPa and temperatures from 373.2 to 453.2 K. In 1998, Aubert^21^ calculated CO_2_ solubility at pressures up to 9.65 MPa using quartz crystal microbalance technique. Next year, Webb et al.^22^ and Sato et al.^23^ evaluated diffusion and solubility of CO_2_ in polymers under high pressures and temperatures. According to their research, the solubilities increased by increasing pressure and decreased by increasing temperature. In 2000, Sato et al.^15^ suggested empirical relations to determine solubility and diffusion coefficient of CO_2_. They considered pressure and temperature as the dependent variables in the range of 1.025–20.144 MPa and 323.15–453.15 K, respectively. They achieved that solubility of CO_2_ in molten state polymers increases by increasing pressure and decreasing temperature. A year later, Hilic et al.^24^ measured solubility of N_2_ and CO_2_ in polystyrene, which considered pressure from 3.05 to 45 MPa and temperature from 338 to 402 K. In addition, an experimental technique with a vibrating-wire force sensor was applied. They got a linear relationship between increasing solubility with increasing pressure and decreasing temperature. In the same year, Sato et al.^25^ calculated solubilities of CO_2_ at the temperature range of 313.15–373.15 K and pressures up to 17.5 MPa. In 2002, Park et al.^26^ studied about CO_2_ solubility in alkanolamine solutions in the values of 40, 60 and 80 °C for temperature and 0.1–50 psia for pressure. They represented a vapor–liquid equilibrium of CO_2_ in these solutions. In the same year, Sato et al.^27^ examined CO_2_ solubility in poly (2,6-dimethyl-1,4-phenylene ether) (PPO) and PS at temperatures of 373.15, 427.15, and 473.15 K and pressures up to 20 MPa. They obtained that solubility of CO_2_ increases with increasing PPO concentration. A year later, in 2003, Hamedi et al.^28^ predicted the adsorption of CO_2_ in various polymers based on a group contribution equation of state (EoS) with input ranges of 283–453 K and 1–200 bar for temperature and pressure, respectively. Their best result was an average absolute relative error (AARE) of 5.5% for polystyrene. In 2006, Li et al.^29^ measured gas solubilities and diffusivities in polylactide at a temperature of 180–200 K and pressures up to 28 MPa using a magnetic suspension balance (MSB). Furthermore, they adopted a theoretical model based on Fick’s second law to extract diffusion coefficients of N_2_ and CO_2_ in polylactide. They obtained that CO_2_ exhibited lower diffusivity than N_2_ at the same temperature. At that year, Nalawade et al.^9^ used SCCO_2_ as a green solvent for processing polymer melts. They earned SCCO_2_ is applicable in many polymerization processes due to its high solubility in polymers. In 2007, Lei et al.^30^ generated buoyancy correlations and Sanchez and Lacombe equation of state to estimate CO_2_ swelling degree, crystallinity, and solubility in polypropylene. They achieved CO_2_ solubility first decreased and then increased with temperature. Two years later, Khajeh et al.^31^ developed intelligent model based on adaptive neuro fuzzy inference system (ANFIS) to predict solubility of CO_2_ in polymers. They used up to 37 data points for different polymers. In 2011, Xu et al.^32^ investigated a theoretical study of solubility correlations of CO_2_ in ether and carbonyl groups of polymers, namely poly(ethylene oxide) (PEO), poly(propylene oxide) (PPO), poly(vinyl acetate) (PVAc), poly(ethylene carbonate) (PEC) and poly(propylene carbonate) (PPC). They showed that the CO_2_ solubility in PPC is higher than other polymers used in their study. Next year, Han et al.^13^ developed continuous reactions and considered economical concepts in SCCO_2_ applications. In 2013, Li et al.^33^ developed an artificial neural network (ANN) to estimate gas solubilities in polymers. Their research demonstrated good agreement between experimental and predicted data using their correlation. At the same year, Minelli and Sarti^34^ measured solubility and permeability of CO_2_ in various glassy polymers by considering diffusion coefficient as a kinetic factor. In 2015, different mathematical and theoretical approaches by Ting and Yuan^10^, Li et al.^7^ and Quan et al.^12^ were studied to estimate CO_2_ properties including solubility. All of them showed that the CO_2_ solubility has direct relation with pressure and reverse relation with temperature. Two years later, Mengshan et al.^8,35^ developed an artificial neural network and artificial intelligence technique based on diffusion theory to predict solubility of CO_2_ and SCCO_2_ in polymers. In 2019, Soleimani et al.^4^ developed decision tree (DT) based smart model for estimating solubility of CO_2_. They used 515 data points with a range of 306–483.7 K for temperature and 1.025–44.41 MPa for pressure. One year later, Li et al.^36^ investigated a comprehensive review of CO_2_ polymer system. They used two types of multi-scaled methods, namely thermodynamic-calculation model and computer simulation to measure CO_2_ solubility in polymers. Their developed model can be utilized in chemistry and chemical industries, such as phase rheological property and polymer self-assembly. In 2022, various experimental, theoretical, and modeling researches have been done in order to measure solubility of CO_2_ and other gases in water-polymer systems. Sun et al.^37^ measured CO_2_ solubility in oil-based and water-based drilling fluids using the sample analysis approach. Their results indicated that the salting-out effect of electrolyte on gas solubility can be increased with increasing the molar concentration of ions. Their study also showed that the errors of CO_2_ solubility in the oil-based and water-based drilling fluids are 6.75% and 3.47%, respectively. Besides, Ushiki et al.^38^ evaluated CO_2_ solubility and diffusivity in polycaprolactone (PCL) performing perturbed-chain statistical associating fluid theory (PC-SAFT) and free volume methods. According to their work, CO_2_ solubility was recognized to conform with Henry’s law, and the PC-SAFT EoS sufficiently described the solubility. Also, Kiran et al.^39^ assessed diffusivity and solubility of CO_2_ and N_2_ in polymers. They used Sanchez-Lacombe EoS in modeling solubility. Furthermore, Ricci et al.^40^ provided a comprehensive theoretical framework for the supercritical sorption and transport of CO_2_ in polymers. In their study, CO_2_ sorption was modelled utilizing data available across the critical region, at different temperatures and pressures up to 18 MPa.

The present research mostly focuses on generating accurate correlations for CO_2_ solubility prediction considering the pressure and temperature of the polymer as input variables. The generated correlations are based on gene expression programming (GEP) technique. A comprehensive databank including of 53 data points for PBS, 43 data points for PBSA and 92 data points for PS polymer is collected^15,20,24,25^. After generating correlations, statistical and graphical error tests are applied to assess the accuracy of the correlations. Likewise, the capability of the represented correlations in predicting the real trend of the CO_2_ solubility with the change of pressure and temperature is appraised. Lately, the leverage approach is performed to detect the outlier data points in the dataset.

## Data collection

In this research, GEP algorithm was implemented to predict the amount of CO_2_ solubility in three different polymers, namely PBS, PBSA, and polystyrene (PS). For this aim, 53 data points for PBS, 43 data points for PBSA, and 92 data points for PS polymer were collected^15,20,24,25^. In this work, pressure and temperature of carbon dioxide were considered as input parameters. A summary of the gathered data points is shown in Table 1. As pointed up in Table 1, extensive ranges of temperature and pressure of CO_2_ are supplied in this study.

**Table 1:** Summary of experimental data points utilized in this work.

Polymer	Pressure (MPa)	Temperature (K)	CO_2_ solubility (g/g)	Number of data
PBS	1.025–20.144	323.15–453.15	0.00876–0.1761	53
PBSA	1.098–20.074	323.15–453.15	0.01184–0.1741	43
PS	2.068–44.41	338.22–473.15	0.00714–0.16056	92

## Correlation development

**Figure 1:**
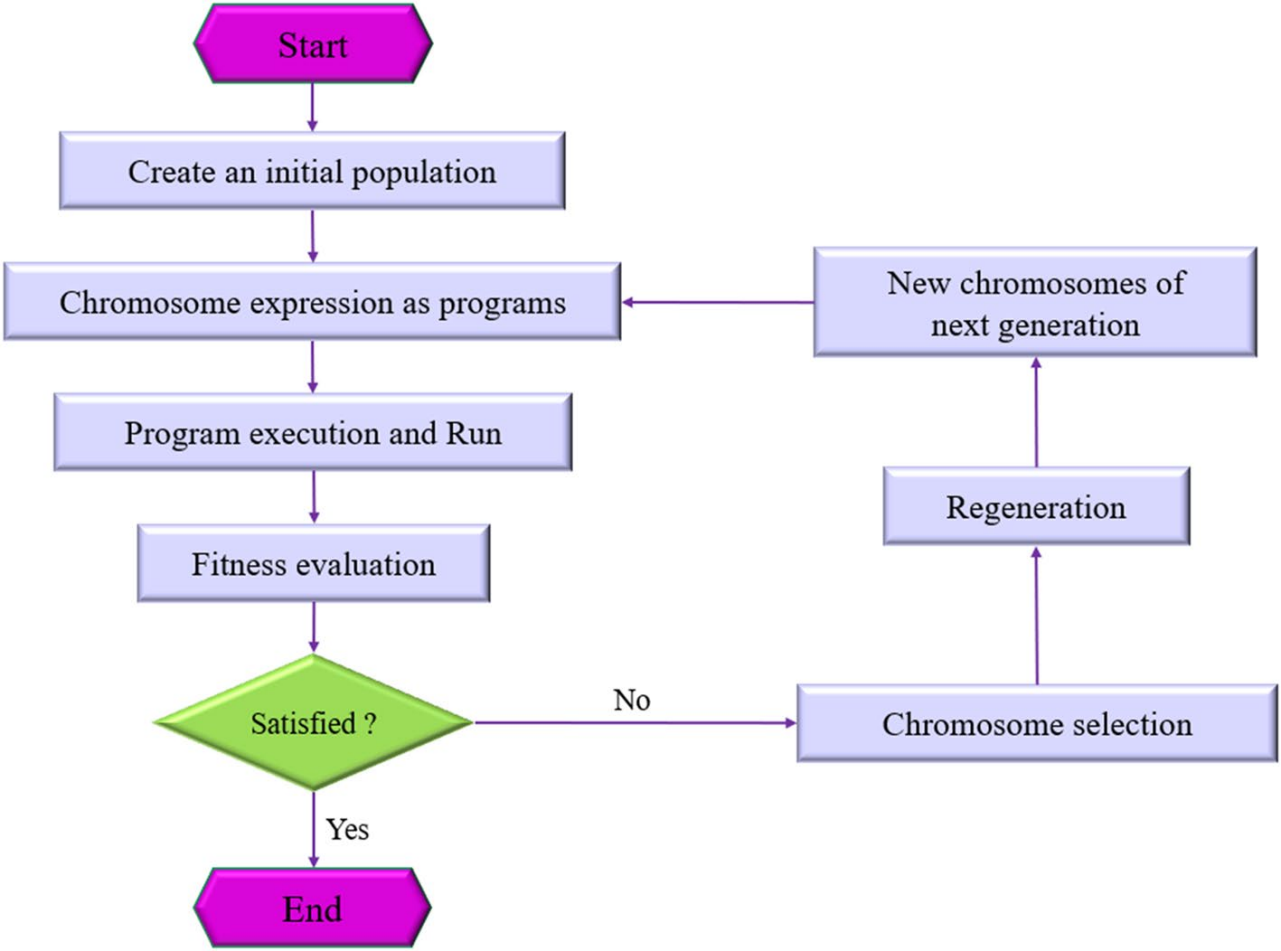
The schematic framework of the gene expression programming (GEP).

In order to generate CO_2_ solubility correlations, Gene expression programming (GEP) evolutionary algorithm has been applied. GEP which was firstly proposed by Ferreira in 2001^41^, is a normally comprehensive phenotype technique in which the chromosomes form a correctly inseparable, operative entity^42^. This technique is extensively used in computer programming and modeling applications^43–46^. Gene expression programming algorithms are complicated tree-based structures that coordinate by changing their shape, composition and sizes. By encoding trees as vectors of symbols and transforming them into them just in order to assess their fitness, this technique can indirectly produce trees^47^. This soft computing technique is strong predictive algorithm that is widely used for various field application purposes. Commonly, the GEP technique has two components, namely chromosome and the expression trees (ETs). The possible solutions are encoded by the chromosomes and is regarded as the linear string with particular length, hence these solutions will be decoded into the real candidate solution termed expression tree^48^. After producing of chromosomes of first-production individuals and choosing them based on fitness function to re-generate with modifications, new generation individuals were presented to the developmental operation of selection environment confrontation, genome expression, and modified reproduce^49^. Additionally, gene expression programming automatically creates algebraic expressions to answer nonlinear problems^50^. The schematic flowchart of GEP procedure is depicted in Fig. 1.

## Results and discussion

### Development of correlations

In the present study, gene expression programming tree-based soft computing approach was carried out to develop accurate correlations for predicting CO_2_ solubility in different polymers. The developed correlations consider CO_2_ solubility as a function of pressure and temperature of corresponding polymer and use them as input variables. To generate accurate and user-friendly correlations, an exhaustive databank consists of 53 data points for PBS polymer, 43 data points for PBSA polymer and 92 data points for PS polymer was collected from previous literature. Table 2 represents the GEP parameters utilized in this research.

**Table 2:** GEP setting parameters used in the study.

Parameters	Value/setting
The number of head size	10–15
Chromosome	500
Number of generation	300
Mutation rate	0.25
Inversion rate	0.1
Operators used	+, −, $\times$, /, exp, X^2^, INV, cos, ln, sqrt

Using the aforementioned approach, the final formulas for the determination of CO_2_ solubility based on gene expression programming technique, are listed below:

#### CO_2_ solubility correlation in PBS polymer

1 $${S}_{{CO}_{2}}=\frac{2.403631\times P\times \left(T+\left(P+2\times T\right)\times \mathit{cos}\left(\mathit{cos}\left(\frac{T}{\mathit{cos}\left(\mathit{cos}\left(\frac{8}{T}\right)\right)}\right)\right)\right)}{T\times \left(P+2\times T\right)}-0.000401$$

#### CO_2_ solubility correlation in PBSA polymer

2 $${S}_{{CO}_{2}}=0.009492+\frac{59525.424282\times \left(\left(P-8\right)\times \left(P+T\right)\times sigmoid\left(P\right)+16\times \left(P-sigmoid\left(P\right)\right)\times \left(T+0.83907\right)\times \mathrm{cos}\left(sigmoid\left(P\right)\right)\right)}{{T}^{2}\times \left(P+T\right)\times \left(T+0.83907\right)\times sigmoid\left(P\right)}$$

#### CO_2_ solubility correlation in PS polymer

3 $${S}_{{CO}_{2}}=\frac{980.876013\times P}{\left(T-sigmoid\left(P\right)\right)\times \left(8\times P+T-72\times sigmoid\left(P\right)-48\right)}+\frac{13.623278\times \mathrm{cos}\left(\mathrm{cos}\left(T\right)\right)}{T\times sigmoid\left(P\right)}-0.040355$$

where P and T denote pressure and temperature of aforenamed polymers, respectively. In the above correlations, the units of P and T are MPa and K, respectively. The generated correlations in this study are applicable for CO_2_ solubility prediction in various ranges of temperature and pressure of the mentioned polymers.

### Statistical performance assessment

In order to show and compare the precision of the generated correlations, some important statistical parameters including root mean square error (RMSE), standard deviation (SD), coefficient of determination (R^2^), the average relative error (ARE) and the average absolute relative error (AARE) were applied^51^. These terms are given below:

4 $$\mathrm{RMSE }= \sqrt{\frac{1}{n} \sum_{i=1}^{n}{ ( S (exp) - S (cal) ) }^{2}}$$

5 $$\mathrm{SD }= \sqrt{\frac{1}{n-1}\sum_{i=1}^{n}{ ( \frac{S \left(exp\right) - S \left(cal\right)}{S (exp)} )}^{2}}$$

6 $$\mathrm{R}^{2} = 1 - \frac{\sum_{i=1}^{n}{ ( S \left(exp\right) -S \left(cal\right) )}^{2} }{\sum_{i=1}^{n}{ ( S \left(cal\right) - S (avg) )}^{2}}$$

7 $$\mathrm{ARE }= \frac{1 }{n} \sum_{i=1}^{n} (Ei)$$

where $Ei$ is the partial deviation that is described as:

8 $$Ei = \left[ \frac{S\left(\mathrm{exp}\right)- S (cal) }{S (exp)} \right] \times 100$$

9 $$\mathrm{AARE }= \frac{1 }{n} \sum_{i=1}^{n} \left|Ei\right|$$

where *n*, *S (exp)*, *S (cal)* and *S (avg)* are the number of data, actual CO_2_ solubility value, calculated CO_2_ solubility value, and the average of the actual data points, respectively. The prementioned statistical parameters for the three generated correlations are detailed for the training, testing, and whole datasets in Table 3. As described in this table, the AARE of the correlation for the PBS polymer is lower than other two correlations generated in this work. Results demonstrate that generated correlation for the PBS polymer has the lowest standard deviation (0.028) and RMSE (0.00178). However, the correlations developed for the other two polymers also have acceptable accuracy. As presented in Table 3, the AARE values for PBS and PBSA polymers were obtained less than AARE for PS polymer, which was due to the nature of the experimental data related to PS polymer. It is obvious that the generated correlations are reliable and sometimes, due to the nature of the experimental data values of different materials (like polymers), different error values may be obtained.

**Table 3:** Statistical assessment of the generated correlations in this work.

Polymer	Status	ARE (%)	AARE (%)	RMSE	SD	R^2^
PBS	Train	0.217	1.775	0.001	0.031	0.997
	Test	0.510	1.097	0.000	0.014	0.999
	All	0.278	1.635	0.001	0.028	0.998
PBSA	Train	1.660	1.271	0.002	0.092	0.995
	Test	− 4.402	8.175	0.004	0.099	0.989
	All	0.391	5.879	0.003	0.093	0.993
PS	Train	3.305	9.197	0.004	0.156	0.991
	Test	4.274	11.819	0.005	0.242	0.973
	All	3.494	9.710	0.004	0.176	0.989

### Graphical performance assessment

**Figure 2:**
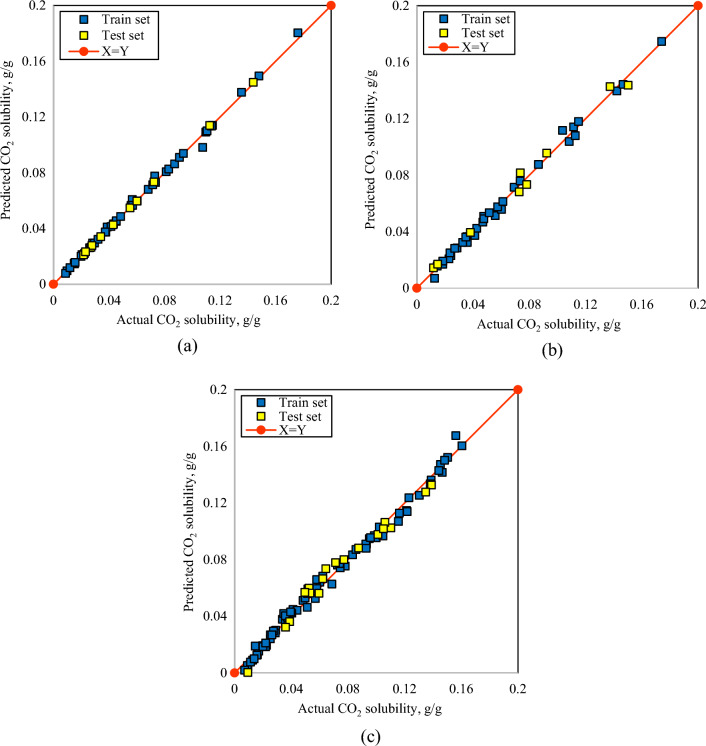
Cross plots of the predicted and experimental CO_2_ solubility values in (**a**) PBS, (**b**) PBSA, (**c**) PS polymers.

**Figure 3:**
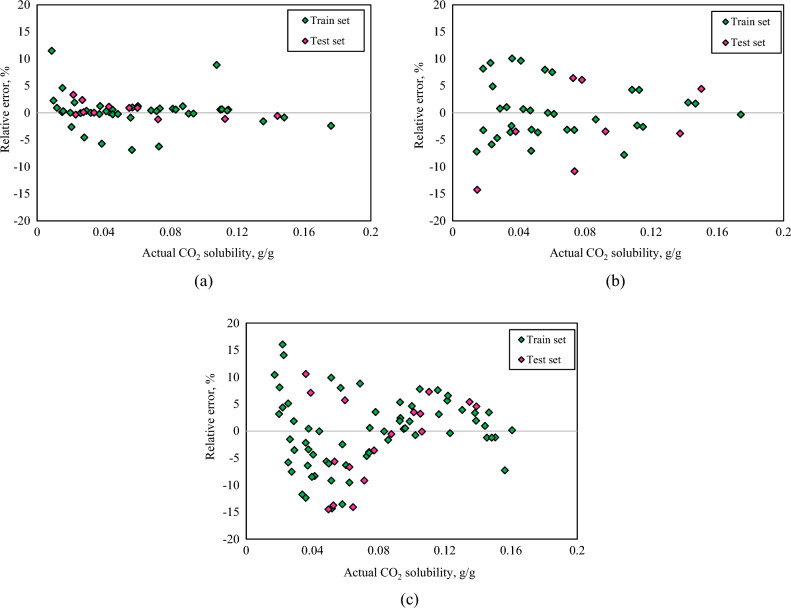
Relative error distribution curves of the generated correlation of the CO_2_ solubility in (**a**) PBS, (**b**) PBSA, (**c**) PS polymers.

This section represents a graphical description of the comparison among the results of the generated correlations and the actual data. The predicted CO_2_ solubility values in PBS polymer are sketched versus actual ones in Fig. 2a. Likewise, the predicted CO_2_ solubility values in PBSA and PS polymers are depicted versus experimental data in Fig. 2b,c, respectively. The closer the sketched data points to the 45° line, the greater the uniformity of the correlations is. According to these plots, it is apparent that the results of the generated user-friendly correlations illustrate satisfactory agreement around the ideal line. Additionally, the relative error curves of the developed correlations of the CO_2_ solubility in PBS polymer, PBSA polymer, and PS polymer are presented in Fig. 3a–c, respectively.

**Figure 4:**
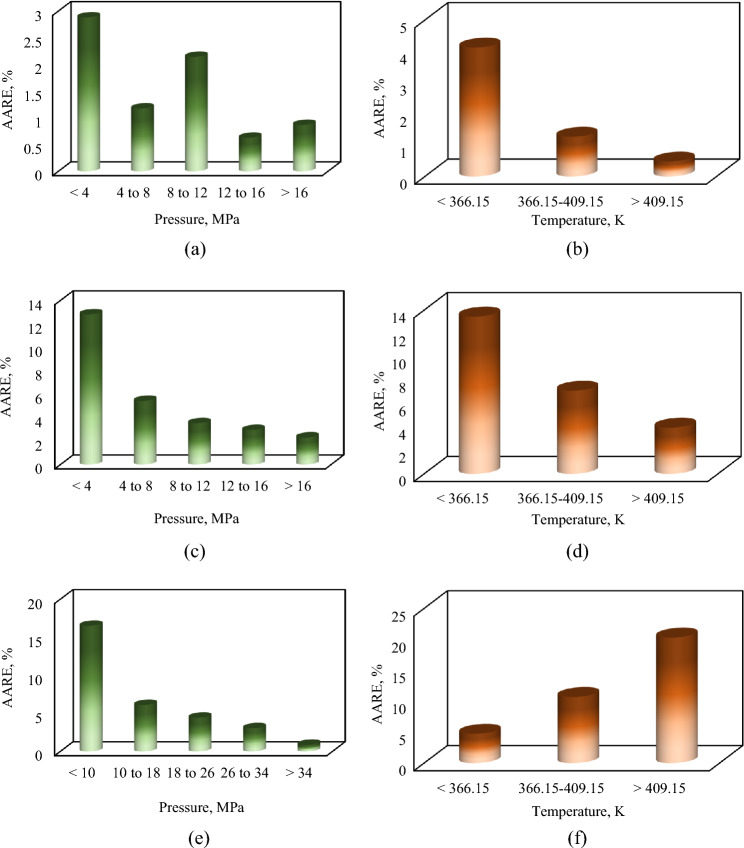
AARE for the different correlations performed in this research for the three polymers in various inputs ranges. (**a**,**b**) PBS; (**c**,**d**) PBSA; (**e**,**f**) PS.

Furthermore, to show the accuracy of the presented correlations in different ranges of pressure and temperature, the correlations’ performances in terms of AARE were sketched against five sets of pressure and three sets of temperature. Figure 4 demonstrates the AARE of the correlations in different ranges of input parameters. For various ranges of pressure, the correlation of CO_2_ solubility in PBS polymer clarifies a steady performance and its AARE is lower than 2.9% in all ranges. Besides, a reliable performance can be perceived from the correlation of CO_2_ solubility in PBS polymer up to the last temperature range. This figure validates the efficiency of the developed correlation of CO_2_ solubility in PBS polymer over other developed correlations in the present study.

**Figure 5:**
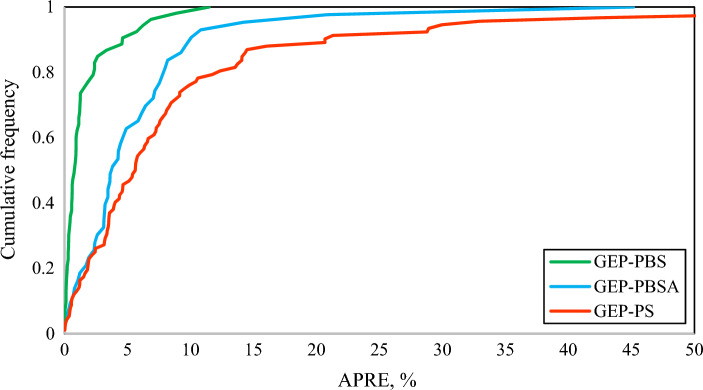
Cumulative frequency plot of generated correlations in this study.

Afterwards, the cumulative frequency analysis of the absolute percent relative error (APRE) for the generated correlations in this work is shown in Fig. 5. According to the results of this figure, the correlation of CO_2_ solubility in PBS polymer could estimate more than 90% of CO_2_ solubility values with an APRE of less than 5%, and also more than 98% of the CO_2_ solubility values by the correlation for PBS polymer have an AARE of less than 10%.

**Figure 6:**
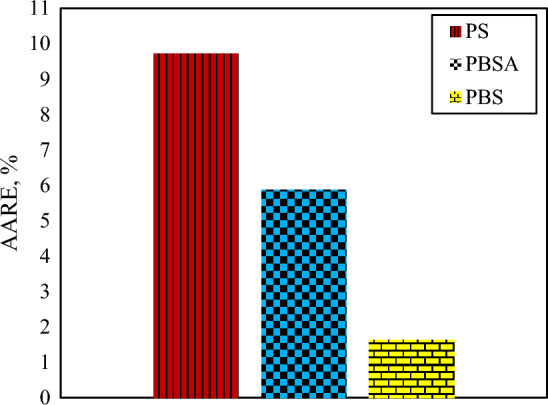
Comparison among AARE values of the implemented correlations.

Additionally, absolute relative error comparison among generated correlations was carried out. Figure 6 describes the AARE comparison between the prementioned correlations. According to this figure, the developed correlation of CO_2_ solubility in PBS polymer revealed the highest accuracy and the lowest AARE between other correlations generated in this research.

### Trend analysis of the generated correlations

**Figure 7:**
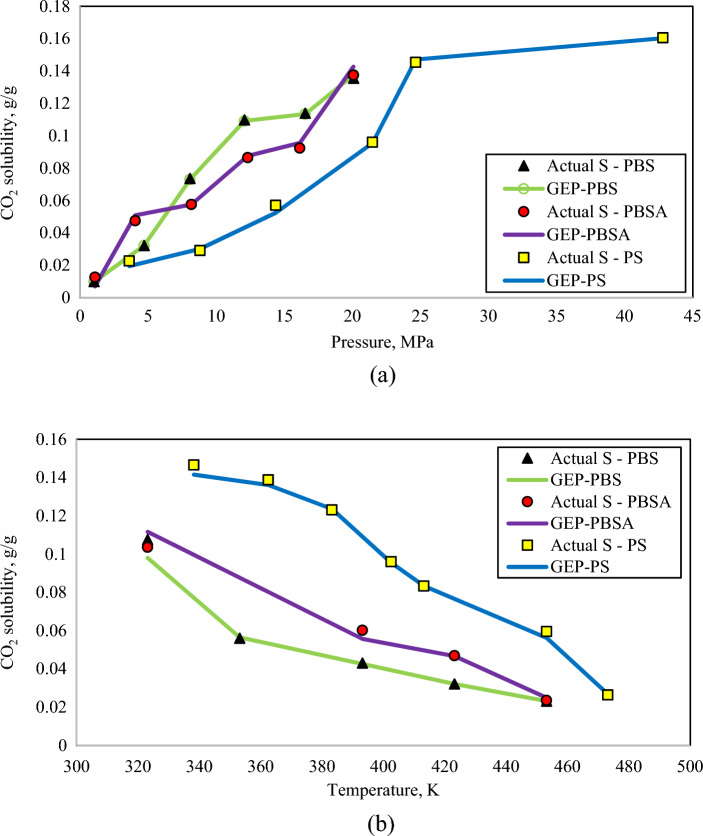
Comparison of the CO_2_ solubility variation for the generated correlations in this work with actual data. (**a**) CO_2_ solubility change with pressure; (**b**) CO_2_ solubility change with temperature.

Trend analysis is a well-known applicable technique to visualize the output variation with the change of input variables^52,53^. The predictions of the CO_2_ solubility correlations are depicted versus temperature and pressure in Fig. 7 to investigate the capability of the generated correlations in following the actual expected trends of CO_2_ solubility values with the change of pressure and temperature. According to Henry’s law, it is evident that CO_2_ solubility increases with decreasing temperature and increasing pressure^54^. Carbon dioxide has a propensity, namely plasticizing effect^55^. It means that the molecules of CO_2_ are pressured in the chains of the polymer as a consequence of increasing pressure, which results in an extension of the pore space within the molecules and, then, for this reason, an addition of their movement^56,57^. This causes it feasible to absorb more gas molecules. Likewise, by decreasing the temperature the CO_2_ molecules obtain lower kinetic energy and they do not have a tendency for releasing from the solution and for staying in a condition with more independence^58^. As a consequence, the solubility would increase.

### Outlier discovery of the developed correlations

**Figure 8:**
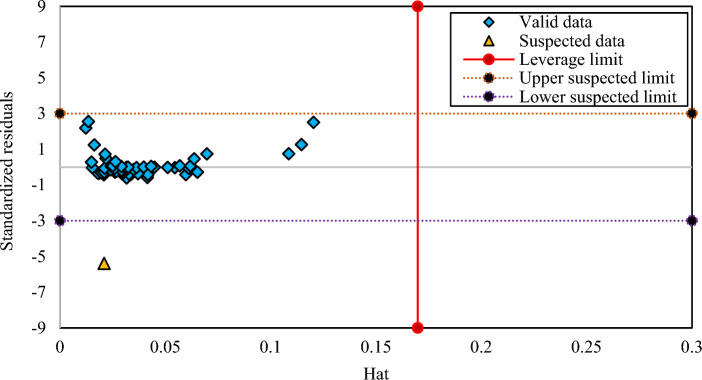
The William plot of the generated correlation for PBS polymer.

**Figure 9:**
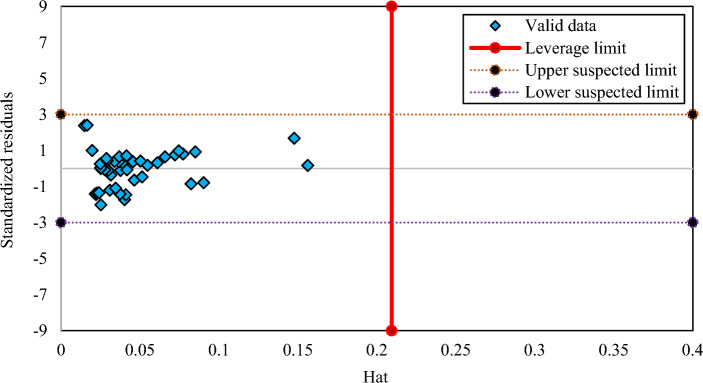
The William plot of the generated correlation for PBSA polymer.

**Figure 10:**
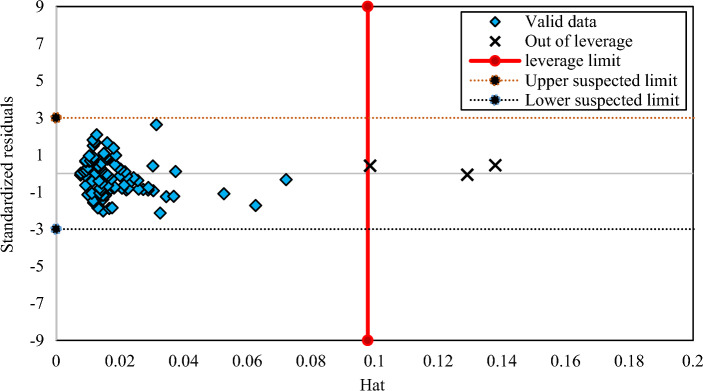
The William plot of the generated correlation for PS polymer.

Outlier discovery plays an important role to identify data that may vary from other data points exist in a dataset^59^. The leverage technique is a trustworthy method for outlier discovery which concerns with the values of the standardized residuals and a matrix, namely the Hat matrix made of the actual and the predicted values obtained from the correlations^60^. According to this approach, if most of the data points located in the ranges of − 3 ≤ R ≤ 3 (R denotes the standardized residual) and 0 ≤ H_i_ ≤ H*, it illustrates that the results of the generated correlations are dependable and valid^61–63^. Figures 8, 9 and 10 represent William plots of the generated correlations of CO_2_ solubility in PBS, PBSA, and PS polymers, respectively. For PBS polymer it is obvious that all of the data points placed in a valid zone except one. Also, the results of the generated correlation of PBSA polymer show that all of the data points located in a valid region. At the end, Fig. 10 presents a William plot of CO_2_ solubility correlation in PS polymer, showing that among whole dataset consists of 92 data points used for this polymer, only 3 data points are recognized as out of leverage data points.

## Conclusions

The present research aimed to predict CO_2_ solubility as a strong effective parameter in polymerization processes. PBS, PBSA, and PS were three polymers, which were utilized in this work. For this purpose, gene expression programming (GEP) technique was applied. To this aim, a widespread dataset was gathered from previous literature. Results showed that the generated correlation of CO_2_ solubility for PBS polymer could present the highest accuracy in predicting solubility of CO_2_ with an AARE of 1.63%, SD of 0.028, and RMSE of 0.001. The sketched CO_2_ solubility curves using the trend analysis demonstrated that all three generated correlations in this study could exactly fit the actual trends of CO_2_ solubility variation. The simple generated correlations can be performed in wide ranges of pressures and temperatures and represent high accuracy. The leverage approach showed that all the data points seem to be reliable and valid except four, which were placed in a lower suspected and out of leverage zones. In order to precisely simulate CO_2_ solubility in polymers in a future works, it is recommended to generate new correlations, and also develop intelligent schemes.

## Data Availability

All the data are available from the corresponding author on reasonable request.

## References

[1] Sheng JJ (2013). Enhanced Oil Recovery Field Case Studies.

[2] Thomas S (2008). Enhanced oil recovery—an overview. Oil Gas Sci. Technol. Rev. l'IFP.

[3] Divandari H, Amiri-Ramsheh B, Zabihi R (2023). Steam flooding (steam drive). Thermal Methods.

[4] Soleimani R (2020). Evolving an accurate decision tree-based model for predicting carbon dioxide solubility in polymers. Chem. Eng. Technol..

[5] Li D-C, Liu T, Zhao L, Yuan W-K (2009). Solubility and diffusivity of carbon dioxide in solid-state isotactic polypropylene by the pressure-decay method. Ind. Eng. Chem. Res..

[6] Zheng H, Mahmoudzadeh A, Amiri-Ramsheh B, Hemmati-Sarapardeh A (2023). Modeling viscosity of CO_2_–N_2_ gaseous mixtures using robust tree-based techniques: Extra tree, random forest, GBoost, and LightGBM. ACS Omega.

[7] Li M (2015). Solubility prediction of supercritical carbon dioxide in 10 polymers using radial basis function artificial neural network based on chaotic self-adaptive particle swarm optimization and K-harmonic means. RSC Adv..

[8] Mengshan L (2017). Prediction of supercritical carbon dioxide solubility in polymers based on hybrid artificial intelligence method integrated with the diffusion theory. RSC Adv..

[9] Nalawade SP, Picchioni F, Janssen L (2006). Supercritical carbon dioxide as a green solvent for processing polymer melts: Processing aspects and applications. Prog. Polym. Sci..

[10] Ru-Ting X, Xing-Yuan H (2015). Predictive calculation of carbon dioxide solubility in polymers. RSC Adv..

[11] Zhang Q, Vanparijs N, Louage B, De Geest BG, Hoogenboom R (2014). Dual pH-and temperature-responsive RAFT-based block co-polymer micelles and polymer–protein conjugates with transient solubility. Polym. Chem..

[12] Quan S (2015). A bio-inspired CO_2_-philic network membrane for enhanced sustainable gas separation. J. Mater. Chem. A.

[13] Han X, Poliakoff M (2012). Continuous reactions in supercritical carbon dioxide: Problems, solutions and possible ways forward. Chem. Soc. Rev..

[14] Chandra R, Rustgi R (1998). Biodegradable polymers. Prog. Polym. Sci..

[15] Sato Y (2000). Solubility and diffusion coefficient of carbon dioxide in biodegradable polymers. Ind. Eng. Chem. Res..

[16] Nishioka M, Tuzuki T, Wanajyo Y, Oonami H, Horiuchi T (1994). Studies in Polymer Science.

[17] Yampolskii Y, Paterson R (2003). Solubility of gases in polymers. Exp. Determin. Solubil..

[18] Shah V, Hardy B, Stern S (1993). Solubility of carbon dioxide, methane, and propane in silicone polymers. Effect of polymer backbone chains. J. Polym. Sci. Part B Polym. Phys..

[19] Li Y-G, Mather AE (1994). Correlation and prediction of the solubility of carbon dioxide in a mixed alkanolamine solution. Ind. Eng. Chem. Res..

[20] Sato Y, Yurugi M, Fujiwara K, Takishima S, Masuoka H (1996). Solubilities of carbon dioxide and nitrogen in polystyrene under high temperature and pressure. Fluid Phase Equilib..

[21] Aubert JH (1998). Solubility of carbon dioxide in polymers by the quartz crystal microbalance technique. J. Supercrit. Fluids.

[22] Webb KF, Teja AS (1999). Solubility and diffusion of carbon dioxide in polymers. Fluid Phase Equilib..

[23] Sato Y, Fujiwara K, Takikawa T, Takishima S, Masuoka H (1999). Solubilities and diffusion coefficients of carbon dioxide and nitrogen in polypropylene, high-density polyethylene, and polystyrene under high pressures and temperatures. Fluid Phase Equilib..

[24] Hilic S, Boyer SA, Pádua AA, Grolier JPE (2001). Simultaneous measurement of the solubility of nitrogen and carbon dioxide in polystyrene and of the associated polymer swelling. J. Polym. Sci. Part B Polym. Phys..

[25] Sato Y, Takikawa T, Takishima S, Masuoka H (2001). Solubilities and diffusion coefficients of carbon dioxide in poly (vinyl acetate) and polystyrene. J. Supercrit. Fluids.

[26] Park SH, Lee KB, Hyun JC, Kim SH (2002). Correlation and prediction of the solubility of carbon dioxide in aqueous alkanolamine and mixed alkanolamine solutions. Ind. Eng. Chem. Res..

[27] Sato Y, Takikawa T, Yamane M, Takishima S, Masuoka H (2002). Solubility of carbon dioxide in PPO and PPO/PS blends. Fluid Phase Equilib..

[28] Hamedi M, Muralidharan V, Lee B, Danner RP (2003). Prediction of carbon dioxide solubility in polymers based on a group-contribution equation of state. Fluid Phase Equilib..

[29] Li G, Li H, Turng L, Gong S, Zhang C (2006). Measurement of gas solubility and diffusivity in polylactide. Fluid Phase Equilib..

[30] Lei Z, Ohyabu H, Sato Y, Inomata H, Smith RL (2007). Solubility, swelling degree and crystallinity of carbon dioxide–polypropylene system. J. Supercrit. Fluids.

[31] Khajeh A, Modarress H, Rezaee B (2009). Application of adaptive neuro-fuzzy inference system for solubility prediction of carbon dioxide in polymers. Expert Syst. Appl..

[32] Xu M, Chen J, Zhang C, Du Z, Mi J (2011). A theoretical study of structure–solubility correlations of carbon dioxide in polymers containing ether and carbonyl groups. Phys. Chem. Chem. Phys..

[33] Li M (2013). Prediction of gas solubility in polymers by back propagation artificial neural network based on self-adaptive particle swarm optimization algorithm and chaos theory. Fluid Phase Equilib..

[34] Minelli M, Sarti GC (2013). Permeability and solubility of carbon dioxide in different glassy polymer systems with and without plasticization. J. Membr. Sci..

[35] Mengshan L, Wei W, Bingsheng C, Yan W, Xingyuan H (2017). Solubility prediction of gases in polymers based on an artificial neural network: A review. RSC Adv..

[36] Li M (2020). Models for the solubility calculation of a CO_2_/polymer system: A review. Mater. Today Commun..

[37] Sun X (2022). Experiments and modeling of CO_2_ solubility in water-based and oil-based drilling fluids. J. Petrol. Sci. Eng..

[38] Ushiki I, Kawashima H, Kihara S-I, Takishima S (2022). Solubility and diffusivity of supercritical CO_2_ for polycaprolactone in its molten state: Measurement and modeling using PC-SAFT and free volume theory. J. Supercrit. Fluids.

[39] Kiran E, Sarver JA, Hassler JC (2022). Solubility and diffusivity of CO_2_ and N_2_ in polymers and polymer swelling, glass transition, melting, and crystallization at high pressure: A critical review and perspectives on experimental methods, data, and modeling. J. Supercrit. Fluids.

[40] Ricci E, De Angelis MG, Minelli M (2022). A comprehensive theoretical framework for the sub and supercritical sorption and transport of CO_2_ in polymers. Chem. Eng. J..

[41] Ferreira, C. Gene expression programming: A new adaptive algorithm for solving problems. *arXiv preprint cs/0102027* (2001).

[42] Umar AA, Saaid IM, Sulaimon AA, Pilus RM (2020). Predicting the viscosity of petroleum emulsions using gene expression programming (GEP) and response surface methodology (RSM). J. Appl. Math..

[43] Zhong J, Feng L, Ong Y-S (2017). Gene expression programming: A survey. IEEE Comput. Intell. Mag..

[44] Amar MN, Larestani A, Lv Q, Zhou T, Hemmati-Sarapardeh A (2022). Modeling of methane adsorption capacity in shale gas formations using white-box supervised machine learning techniques. J. Petrol. Sci. Eng..

[45] Amar MN (2021). Prediction of hydrate formation temperature using gene expression programming. J. Nat. Gas Sci. Eng..

[46] Amar MN, Ghriga MA, Seghier MEAB, Ouaer H (2021). Predicting solubility of nitrous oxide in ionic liquids using machine learning techniques and gene expression programming. J. Taiwan Inst. Chem. Eng..

[47] Baniasadi H, Kamari A, Heidararabi S, Mohammadi AH, Hemmati-Sarapardeh A (2015). Rapid method for the determination of solution gas-oil ratios of petroleum reservoir fluids. J. Nat. Gas Sci. Eng..

[48] Rostami A, Arabloo M, Kamari A, Mohammadi AH (2017). Modeling of CO_2_ solubility in crude oil during carbon dioxide enhanced oil recovery using gene expression programming. Fuel.

[49] Mirzaie M, Tatar A (2020). Modeling of interfacial tension in binary mixtures of CH_4_, CO_2_, and N_2_-alkanes using gene expression programming and equation of state. J. Mol. Liq..

[50] Traore S, Luo Y, Fipps G (2017). Gene-expression programming for short-term forecasting of daily reference evapotranspiration using public weather forecast information. Water Resour. Manage.

[51] Sarapardeh AH, Larestani A, Menad NA, Hajirezaie S (2020). Applications of Artificial Intelligence Techniques in the Petroleum Industry.

[52] Kirk H, Haynes F, Monroe R (1980). Application of trend analysis to horticultural field trials. J. Am. Soc. Hortic. Sci..

[53] Amiri-Ramsheh B, Safaei-Farouji M, Larestani A, Zabihi R, Hemmati-Sarapardeh A (2022). Modeling of wax disappearance temperature (WDT) using soft computing approaches: Tree-based models and hybrid models. J. Petrol. Sci. Eng..

[54] Kumełan J, Kamps AP-S, Tuma D, Maurer G (2006). Solubility of CO_2_ in the ionic liquid [hmim][Tf2N]. J. Chem. Thermodyn..

[55] Minelli M, Oradei S, Fiorini M, Sarti GC (2019). CO_2_ plasticization effect on glassy polymeric membranes. Polymer.

[56] Khoshraftar Z, Ghaemi A (2023). Prediction of CO_2_ solubility in water at high pressure and temperature via deep learning and response surface methodology. Case Stud. Chem. Environ. Eng..

[57] Messabeb H, Contamine F, Cézac P, Serin JP, Gaucher EC (2016). Experimental measurement of CO_2_ solubility in aqueous NaCl solution at temperature from 323.15 to 423.15 K and pressure of up to 20 MPa. J. Chem. Eng. Data.

[58] Thibault Y, Holloway JR (1994). Solubility of CO 2 in a Ca-rich leucitite: Effects of pressure, temperature, and oxygen fugacity. Contrib. Miner. Petrol..

[59] Rousseeuw PJ, Van Zomeren BC (1990). Unmasking multivariate outliers and leverage points. J. Am. Stat. Assoc..

[60] Rousseeuw PJ, Leroy AM (2005). Robust Regression and Outlier Detection.

[61] Goodall, C. R. 13 Computation using the QR decomposition. (1993).

[62] Gramatica P (2007). Principles of QSAR models validation: Internal and external. QSAR Comb. Sci..

[63] Amiri-Ramsheh B, Zabihi R, Hemmati-Sarapardeh A (2023). Modeling wax deposition of crude oils using cascade forward and generalized regression neural networks: Application to crude oil production. Geoenergy Sci. Eng..

